# Responses of Soil Microbial Communities and Anthracnose Dynamics to Different Planting Patterns in *Dalbergia odorifera*

**DOI:** 10.3390/microorganisms13122876

**Published:** 2025-12-18

**Authors:** Long Xu, Kexu Long, Yichi Zhang, Guoying Zhou, Junang Liu

**Affiliations:** 1 Key Laboratory for Non-Wood Forest Cultivation and Conservation of Ministry of Education, Central South University of Forestry and Technology, Changsha 410004, China; 15922410904@163.com (L.X.); lkx15675416974@163.com (K.L.); zhangyichi0103111@126.com (Y.Z.); zgyingqq@163.com (G.Z.); 2 Hunan Provincial Key Laboratory for Control of Forest Diseases and Pests, Central South University of Forestry and Technology, Changsha 410004, China; 3 The National Key Laboratory of Woody Oil Resources Utilization, Central South University of Forestry and Technology, Changsha 410004, China; 4 Yuelushan Laboratory Non-Wood Forests Variety Innovation Center, Central South University of Forestry and Technology, Changsha 410004, China

**Keywords:** *Dalbergia odorifera*, anthracnose, planting patterns, soil microbial community, ecological disease suppression, functional network

## Abstract

Anthracnose is one of the major diseases affecting *Dalbergia odorifera* T. Chen. However, the soil microbial mechanisms underlying *D. odorifera* responses to anthracnose remain largely unexplored. This study investigated three planting systems: a *Dalbergia odorifera* monoculture (J); a mixed plantation of *D. odorifera* and *Pterocarpus macrocarpus* (JD); and a composite mixed plantation of *D. odorifera*, *P. macrocarpus*, and *Clinacanthus nutans* (JDY). Using amplicon sequencing technology for soil microbial analysis and combining soil physical and chemical properties with disease severity, we comprehensively analyzed changes in soil microbial community structure and function across different planting modes. The results showed that the diverse mixed mode (JD, JDY) significantly improved soil physicochemical properties and promoted soil nutrient cycling. Redundancy analysis (RDA) indicated that soil organic matter (SOM) and disease severity, quantified by the area under the disease progress curve (AUDPC), were the primary environmental drivers of microbial community variation. Genera positively correlated with SOM and negatively correlated with AUDPC were significantly enriched in JDY and JD, whereas genera showing opposite relationships were predominantly enriched in J. Functional predictions revealed enhanced nutrient-cycling capacities in JD and JDY, with JDY uniquely harboring functional groups such as Arbuscular Mycorrhizal, Epiphyte, and Lichenized taxa. In contrast, microbial functions in the J plantation were mainly limited to environmental amelioration. Co-occurrence network analysis further showed that as planting patterns shifted from J to JDY, microbial communities evolved from competition-dominated networks to cooperative defensive networks, integrating efficient decomposition with strong pathogen suppression potential. The study demonstrates that complex mixed planting systems regulate soil properties, enhance the enrichment of key functional microbial taxa, reshape community structure and function, and ultimately enable ecological control of anthracnose disease. This study provides new perspectives and theoretical foundations for ecological disease management in plantations of rare tree species and for microbiome-based ecological immunization strategies.

## 1. Introduction

*Dalbergia odorifera* T. Chen, commonly known as Hainan huanghuali, is a semi-evergreen tree of the genus *Dalbergia* (Fabaceae) and is listed as a nationally protected Class II rare and endangered species in China [1]. Owing to its exceptional wood quality, *D. odorifera* is widely used in high-end furniture, delicate handicrafts, valuable musical instruments, and decorative artwork. Its heartwood is listed in the official Chinese Pharmacopoeia and exhibits notable pharmacological properties, including therapeutic effects on cardiovascular diseases and potent antioxidant, anti-inflammatory, and antitumor activities [2,3]. Anthracnose, caused by *Colletotrichum* spp., typically results in characteristic black necrotic lesions on leaves, stems, and fruits, accompanied by premature defoliation and fruit rot. Severe infections can lead to plant decline and substantial yield losses [4]. In recent years, anthracnose outbreaks have occurred frequently across major *D. odorifera*-producing regions in southern China. Several *Colletotrichum* pathogens have been identified on *D. odorifera*, including *C. brevisporum*, *C. gigasporum*, and *C. karstii*. These pathogens infect hosts via conidia, producing brown to grayish-white lesions on leaves, young shoots, and fruits. Dark acervuli or orange conidial masses are often visible at lesion centers, with dark concentric rings and occasionally yellow halos at the margins. Lesions commonly develop along leaf margins, and severe infections cause extensive leaf blight, defoliation, and even whole-plant mortality. Such damage suppresses the growth of young *D. odorifera* trees, deteriorates wood quality, and leads to substantial economic loss [1,5,6]. Currently, *D. odorifera* plantations predominantly employ monoculture systems. Although monocultures facilitate short-term management and yield control, long-term implementation often leads to impoverished understory vegetation, increased risk of disease transmission, and reduced ecosystem stability [7,8]. Moreover, intensified intraspecific competition within monocultures suppresses vigorous tree growth and high-quality wood formation [7], imposing significant constraints on the sustainable development of the *D. odorifera* industry [9].

Diversified mixed-planting systems offer pronounced advantages in ecological stability and disease suppression. The interactions include various types, such as competition, mutualistic symbiosis, facilitation, and antagonism. Among them, mutualistic symbiosis, promoting effects, and “dilution effects” are of great significance in inhibiting pathogens in diverse communities [10]. Diversified planting reduces the spread of pathogens between single hosts through ecological niche complementarity and resource allocation, forming a natural “dilution effect” that effectively reduces pathogen spread and colonization and significantly reduces the incidence of fungal diseases [11,12], and effectively suppresses disease outbreaks [13]. Different planting patterns alter litter composition and root exudation (e.g., organic acids, phenolics), thereby modulating soil C, N, and P cycling and associated enzyme activities (e.g., urease, phosphatase, chitinase), ultimately reshaping microbial community structure [14]. Root exudates are chemically complex, comprising organic acids, sugars, phenolics, flavonoids, aliphatic compounds, and secondary metabolites [15]. Their composition and relative abundance vary substantially across plant species, genotypes, developmental stages, and environmental conditions [16]. *Rhizobium* acts as a key regulatory microorganism in legume rhizospheres, supplying nitrogen through symbiotic fixation and enhancing plant growth and biomass accumulation [17]. Beyond increasing nitrogen use efficiency, *Rhizobium* secretes diverse signaling molecules—such as IAA, Nod factors, and ACC deaminase—to regulate root development, induce systemic resistance, promote beneficial microbial colonization, and indirectly suppress pathogens [18]. It can also further enhance plant resilience to stress and disease through multiple mechanisms, including phosphate solubilization, siderophore production, and the secretion of antimicrobial compounds [19]. Plant–plant interactions modulate rhizosphere microbial communities and their gene expression by altering root exudation profiles. For instance, in a *Solanum lycopersicum*–*Allium cepa* intercropping system, flavonoids (e.g., taxifolin) secreted by *A. cepa* roots promote the enrichment of *Bacillus* in the tomato rhizosphere, thereby enhancing resistance to *Fusarium* wilt [20]. Root exudates not only shift microbial community composition but also regulate the expression of genes involved in metabolism, transport, regulation, and stress responses in beneficial bacteria, such as *Pseudomonas*, thereby reinforcing their antagonistic activity against pathogens [15]. Additionally, *Rhizobium*–legume symbiosis modulates the expression of functional genes in rhizosphere microbiomes through signaling molecules, thereby enhancing nitrogen fixation, phosphate solubilization, and disease resistance [19]. Mixed planting of *D. odorifera* with other tree species optimizes nutrient cycling and rhizosphere interactions, improves stand structure and microbial stability, and effectively suppresses pathogen spread and infection [21]. Mixed planting of *D. odorifera* with *Santalum album* L. significantly enhances nitrogen mineralization and reduces nitrogen leaching by more than 25% [22], while intercropping with *Eucalyptus* spp. increases soil microbial activity and nitrogen-cycling enzyme activity, thereby improving nitrogen uptake and biomass accumulation [23].

Soil microorganisms serve as essential regulators and executors in maintaining plant health [24]. Microbe-induced systemic resistance (ISR) has become increasingly recognized for its crucial role in protecting plants against pathogens. Beneficial bacteria, such as *Bacillus*, secrete lipopeptides (e.g., surfactin and fengycin) that can directly suppress pathogens and activate plant defense systems [25]. These lipopeptides enhance broad-spectrum resistance by modulating plant defense signaling pathways (e.g., jasmonic acid and ethylene) and regulating the expression of multiple defense-related genes [26]. Arbuscular mycorrhizal fungi (AMF) form symbiotic associations with plant roots, improving nutrient and water uptake while inducing ISR to enhance host health [27,28]. Diversified planting communities drive the reshaping of microbial community structure and functional networks by changing resource inputs and microenvironmental conditions, thereby forming key functional microbial alliances with specific ecological functions [29]. Environmental factors act as functional filters, playing pivotal roles in microbial community assembly and ecosystem stability [30]. The mechanism of “environmental filtering—community restructuring—network stability—ecological immunity” is regarded as a key biological process for maintaining the health and disease resistance of forest ecosystems. [31]. Environmental gradients—such as soil pH, nutrient status, and vegetation structure—shape microbial community assembly and ecological functions through functional filtering [14]. Soil microbial communities not only reflect ecosystem conditions but also actively drive ecological restoration and disease suppression [29].

At present, no studies have reported how soil microbial communities in tropical plantations of the rare tree species *D. odorifera* respond to pathogens under different planting patterns. Therefore, this study focused on *D. odorifera* plantations with varying planting patterns, integrating soil physicochemical properties, anthracnose disease severity, and high-throughput sequencing data to assess changes in soil microbial community structure and function comprehensively. Based on this objective, we hypothesize that planting patterns drive functional reassembly of microbial communities by altering soil properties, and that the enrichment of key responsive taxa mediates ecological suppression of diseases. The study aims to elucidate how different planting patterns influence soil physicochemical properties, thereby reshaping microbial community structure and function to achieve ecological suppression of anthracnose. The findings are expected to provide novel insights for ecological disease management in plantations of rare tree species and offer both theoretical foundations and practical guidance for harnessing microbial community reassembly to achieve ecological immunity.

## 2. Materials and Methods

### 2.1. Study Site and Sample Plot Selection

This study was conducted in the state-owned Chengmai Forest Farm, Chengmai County, China (19°67′ N, 110°01′ E). The region has a typical tropical monsoon climate, with an annual mean temperature of 24.8 °C and an annual precipitation of approximately 149 mm (https://www.weather.com.cn). Based on species diversity gradients, three representative *D. odorifera* plantation sites were selected (Table 1). The stand age of *D. odorifera* in all selected plots was 12 years. The soil is red and has a sandy loam texture. All sites were managed uniformly throughout the study period.

### 2.2. Anthracnose Disease Survey and Soil Sample Collection

**Disease survey:** The investigation began in January 2024. In each plot, the five-point sampling method was used, with standard trees randomly selected in the east, south, west, north, and central areas. All selected trees were tagged and numbered for subsequent monitoring. From January to December 2024, the incidence of anthracnose disease was surveyed monthly. Disease severity was recorded according to standardized grading criteria. The disease incidence and disease index (DI) were calculated for each plot using the methods described by [32,33]. Anthracnose disease severity was classified as follows: Grade 0, no symptoms; Grade 1, lesions covering less than 20% of the leaf area; Grade 2, lesions covering 20–50% of the leaf area; Grade 3, lesions covering 51–75% of the leaf area; Grade 4, lesions covering 76–100% of the leaf area.

The area under the disease progress curve (AUDPC) was used to quantify the overall severity of anthracnose [34]. This index integrates disease information across the entire observation period, providing a more reliable measure of cumulative disease pressure than single-point assessments, and has been widely applied in studies of plant–microbe interactions [35,36]. The AUDPC was calculated as follows:

(1) $$AUDPC=\sum_{i=1}^{n-1}[\frac{\left(y_{i}+y_{i+1}\right)}{2}\times (t_{i+1}-t_{i})]$$

*n* = total number of surveys; i = the i-th survey (starting from 1); y_i_ = disease severity recorded during the i-th survey; t_i_ = time of the i-th survey.

**Soil sampling:** In November 2024, four 5 m × 5 m plots were established within each site where disease surveys had been conducted. Sterilized soil augers (3.5 cm in diameter) were used to randomly collect four soil cores (0–20 cm depth) within each quadrat. The four cores from the same quadrat were thoroughly mixed to form one composite sample. The collected soil samples were placed in sterile bags, homogenized, immediately stored on dry ice, and rapidly transported to the laboratory. One sample was stored at −80 °C for subsequent soil microbial DNA extraction and high-throughput sequencing analysis [37]. In contrast, another sample was air-dried in sterile Petri dishes, sieved through a 2 mm mesh, and used for soil physicochemical analyses. The experiment included three plots, each with four replicates, for a total of 12 soil samples across all three plots.

### 2.3. Soil High-Throughput Sequencing

The collected soil samples were subjected to high-throughput sequencing by Novogene Co., Ltd. (Beijing, China). Total microbial DNA was extracted from soil using the FastDNA^®^ SPIN Kit for Soil (MP Biomedicals, Irvine, CA, USA) following the manufacturer’s protocol. DNA concentration and purity were determined using a NanoDrop^®^ ND-2000 spectrophotometer (Thermo Scientific, Waltham, MA, USA) [38]. DNA libraries were constructed using the NEBNext Ultra II DNA Library Prep Kit (Cat. No. E7645B; New England Biolabs, Ipswich, MA, USA).

The V4 region of the 16S rRNA gene was amplified using primers 515F (5′-GTGCCAGCMGCCGCGGTAA-3′) and 806R (5′-GGACTACHVGGGTWTCTAAT-3′) for bacterial community analysis [39]. The ITS1 region was amplified using primers 1737F (5′-GGAAGTAAAAGTCGTAACAAGG-3′) and 2043R (5′-GCTGCGTTCTTCATCGATGC-3′) for fungal community profiling [40]. PCR amplification was performed in a 30 μL reaction containing 15 μL Phusion High-Fidelity PCR Master Mix, 0.2 μM of each primer, and 10 ng of genomic DNA template. The PCR cycling parameters included an initial denaturation at 98 °C for 1 min, followed by 30 cycles of 98 °C for 10 s, 50 °C for 30 s, and 72 °C for 30 s, with a final extension at 72 °C for 5 min. PCR amplicons were purified using magnetic beads and pooled in equimolar concentrations. Using the Illumina NovaSeq 6000 platform for PE250 mode sequencing with NovaSeq 6000 [41].

### 2.4. Soil Physicochemical Property Analysis

Soil physicochemical parameters were determined as follows: pH was measured using a potentiometric method with a soil-to-water ratio of 1:2.5; soil organic matter (SOM) by the potassium dichromate oxidation–external heating method; total nitrogen (TN) by the Kjeldahl method; available nitrogen (AN) by the alkali diffusion method; total potassium (TK) by the sodium hydroxide fusion–flame photometric method; available potassium (AK) by the ammonium acetate extraction–flame photometric method; total phosphorus (TP) by the sodium hydroxide fusion–molybdenum–antimony colorimetric method; and available phosphorus (AP) by the sodium bicarbonate extraction–molybdenum–antimony colorimetric method. Each soil sample was analyzed in four replicates, and the mean values were used for further statistical analysis [42].

### 2.5. Bioinformatics Analysis

Raw sequencing reads were quality-filtered using Fastp (version 0.23.1) and merged with FLASH (version 1.2.11). Denoising and amplicon sequence variant (ASV) clustering were performed using the DADA2 plugin in QIIME2 (version 2022.11) [43]. The filtering criteria were as follows: (i) sequences shorter than 15 bp or containing more than five ambiguous bases (N) were removed; (ii) sequences with more than 40% of bases having a quality score below Q15 were trimmed, and those shorter than 15 bp after trimming were discarded. Taxonomic assignments were conducted using the Silva database (version 138.1) for 16S rRNA genes [44] and the UNITE database (version 9.0) for ITS sequences [45]. High-throughput sequencing data analysis was conducted on the Novogene cloud platform in Beijing (https://magic-plus.novogene.com) (accessed on 26 September 2025). To minimize noise from extremely rare taxa, only genera with a mean relative abundance ≥ 0.01% and occurring in at least two samples were retained for downstream analyses [46,47]. To identify microbial taxa responding to environmental gradients during community reassembly, redundancy analysis (RDA) was applied to assess associations between bacterial/fungal communities and environmental variables, including soil physicochemical properties and AUDPC. Genera were then ranked based on their combined contributions along the RDA1 and RDA2 axes (i.e., their explanatory power in ordination space), and the top 25 taxa were defined as “key genera” [48,49] for subsequent functional prediction, correlation analyses, and co-occurrence network construction.

### 2.6. Statistical Analysis

Alpha diversity indices (coverage, Chao1, Simpson, and Shannon) were compared among groups using one-way analysis of variance (ANOVA) followed by Tukey’s HSD post hoc test, with a significance threshold of *p* < 0.05. Beta diversity of microbial communities was assessed using Principal Coordinates Analysis (PCoA) based on Bray–Curtis distance matrices, and statistical significance of group differences was tested via permutational multivariate analysis of variance (PERMANOVA). Redundancy analysis (RDA) was conducted to explore the constraining effects of environmental factors on microbial community composition. A microbial co-occurrence network was constructed using Spearman’s correlation coefficient, with selection criteria set at |r| ≥ 0.3 and *p* < 0.05. Network topology parameters were calculated using the igraph package (v1.5.1) and evaluated through node degree, betweenness centrality, average clustering coefficient, and modularity index. Functional potentials of bacterial and fungal communities were predicted and annotated using the FAPROTAX (version 1.2.4) and FUNGuild databases (version 1.2), respectively. Differences in microbial genus-level abundances among treatments were analyzed using the Kruskal–Wallis nonparametric test (*p* < 0.05). For genera showing significant differences (*p* < 0.05), Dunn’s post hoc multiple comparisons were performed to identify specific treatment contrasts. Significance of differences among groups was further evaluated using one-way ANOVA followed by Tukey’s HSD test, with a threshold of *p* < 0.05. All statistical analyses were performed in the R environment (v4.5.2).

## 3. Results

### 3.1. Differences in Anthracnose Severity and Soil Physicochemical Characteristicss

**Figure 1:**
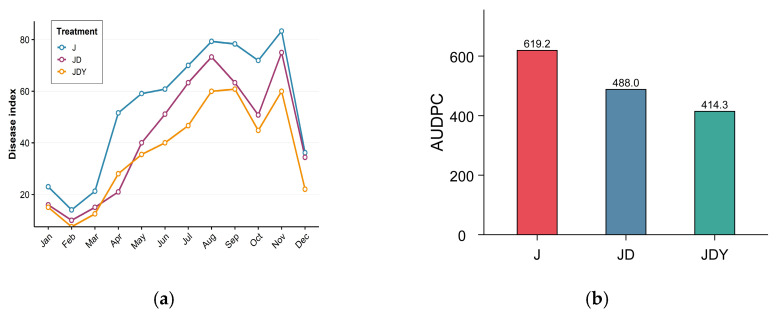
Anthracnose disease severity index curves and area under disease progress curves (AUDPC) of *D. odorifera* plantations under three different planting patterns. (**a**) Disease index curves show monthly changes in anthracnose severity. Data points represent the disease index values from monthly surveys. (**b**) AUDPC reflects the overall severity of the disease, with lower values indicating better disease control. Data are from monthly anthracnose surveys from January to December 2024.

The severity of anthracnose varied among different planting patterns (Figure 1). Both the JDY and JD planting patterns exhibited lower anthracnose severity compared to the J pattern. Disease severity was highest in the J pattern, with the maximum AUDPC values. In contrast, the JDY pattern exhibited the lowest disease severity and AUDPC values, and the JD pattern showed intermediate severity.

Significant differences in soil physicochemical properties were observed among *D. odorifera* plantations under different planting patterns. Soil physicochemical parameters in the JDY and JD patterns were significantly higher than those in the J pattern. Among the three patterns, the J pattern exhibited the lowest values for all measured parameters, with more substantial soil acidity and lower fertility. The JD pattern showed optimal values for several key parameters, including pH, TN, TK, AK, and AP. The JDY pattern exhibited the highest values for SOM, AN, and TP (Table 2).

### 3.2. Effects of Planting Patterns on Soil Microbial Community Structure

Alpha diversity indices of soil microbial communities under the three planting patterns were evaluated (Table 3). The community coverage across all samples reached 1, indicating that the sequencing results were sufficient to capture the microbial diversity in the study samples. For bacterial communities, the JD treatment showed the highest Chao, Simpson, and Shannon indices, both significantly higher than those in the J and JDY treatments. In contrast, fungal diversity exhibited no consistent trend among planting patterns: the Chao index was highest in JD, the Shannon index was highest in J, and the Simpson index peaked in JDY. These results suggest that fungal communities responded in a more complex manner, with distinct response mechanisms compared with those of bacteria.

**Figure 2:**
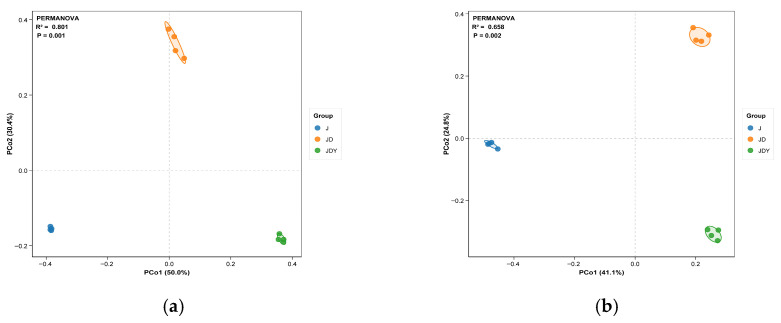
Principal Coordinates Analysis (PCoA) based on ASV-level bacterial (**a**) and fungal (**b**) community composition, showing overall differences in community structure among different planting patterns. The R^2^ and *p* values shown in the figure were obtained from PERMANOVA to assess statistical significance.

Beta diversity was assessed using Principal Coordinates Analysis (PCoA) based on Bray–Curtis distance matrices at both ASV and genus levels. The analysis further confirmed that planting patterns had significant effects on soil microbial community composition, with all three patterns markedly reshaping community structure (Figure 2 and Figure S1). The three planting modes showed significant separation at different levels, and the PERMANOVA test showed highly significant differences between groups (ASV level: bacteria: R^2^ = 0.801, *p* = 0.001; Fungi: R^2^ = 0.658, *p* = 0.002; Genus level: bacteria: R^2^ = 0.823, *p* = 0.001; Fungi: R^2^ = 0.782, *p* = 0.001). These results suggest that planting patterns may drive microbial community assembly.

### 3.3. Effects of Environmental Factors (Including AUDPC) on Soil Microbial Community Structure and Identification of Key Microbial Taxa

**Figure 3:**
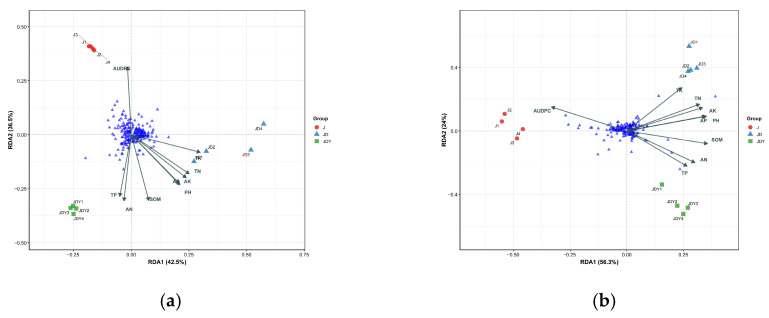
RDA of environmental factors (including AUDPC) and soil microbial communities. (**a**) Community-level RDA of soil bacteria; (**b**) Community-level RDA of soil fungi. The longer the arrow, the stronger the explanatory power, indicating a greater influence of the variable on the RDA space. Conversely, shorter arrows indicate weaker explanatory power. The angle between two arrows reflects their correlation. An angle of 0° represents a perfect positive correlation, 90° represents no correlation, and 180° represents a perfect negative correlation. The blue triangle indicates genus information.

Redundancy analysis (RDA) of soil microorganisms revealed significant differences among planting patterns and among environmental factors (Figure 3). The correlation between microbial community structure under different planting patterns and environmental factors was evaluated using quadrant distributions, arrow lengths, and angles between microbial taxa and environmental factors. As shown in the figure, the selected environmental factors explained 79% of the variation in bacterial community composition affected by planting patterns (RDA1 accounted for 42.5%, RDA2 for 36.5%) and 80.3% of the variation in fungal community composition (RDA1 accounted for 56.3%, RDA2 for 24%). The main environmental factors affecting bacterial community structure were SOM, AUDPC, and pH, in order. The main environmental factors affecting fungal community structure were SOM, TK, AUDPC, and AK, in order. Notably, the AUDPC vector points in the opposite direction to the distribution of JD and JDY samples, aligns with the clustering area of J samples, and forms large obtuse angles with vectors of soil health indicators such as SOM. This intuitively indicates that poorer soil health is associated with higher disease occurrence.

**Figure 4:**
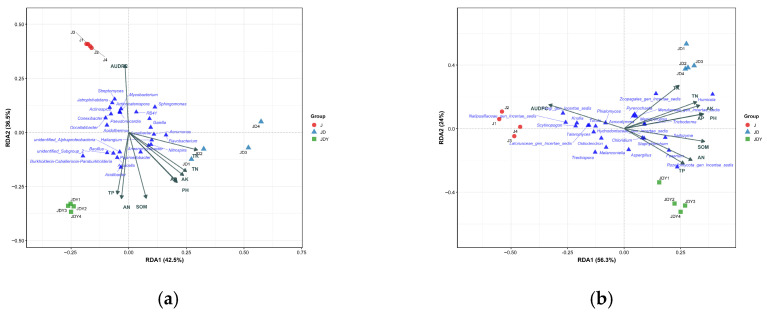
Redundancy analysis (RDA) of environmental factors (including AUDPC) and key microbial groups. (**a**) RDA of key soil bacterial microorganisms at the community level; (**b**) RDA of key soil fungal microorganisms at the community level. Arrows indicate the weight of the corresponding variables. Longer arrows indicate stronger explanatory power, meaning the variable has a greater effect on the RDA space; shorter arrows indicate weaker explanatory power. The angle between two arrows reflects their correlation. 0° indicates a perfect positive correlation, 90° indicates no correlation, and 180° indicates a perfect negative correlation. The blue triangle indicates genus information.

To further identify the microbial taxa exerting the most significant influence during community shifts, the top 25 bacterial and fungal genera with the highest combined contributions to the RDA axes were selected, based on the 79% and 80.3% of community variation explained by the RDA models (Figure 4). The spatial distribution of these key taxa in the RDA ordination clearly reflected their close associations with specific environmental gradients. The five genera with the highest cumulative contributions exhibited distinct planting-pattern preferences: *Burkholderia-Caballeronia-Paraburkholderia*, *Acidibacter*, and *Rozellomycota_gen_Incertae_sedis* were associated with the JDY plots; *Flavobacterium*, *Humicola*, and *Zoopagales_gen_Incertae_sedis* were associated with JD; whereas *Streptomyces*, *Fungi_gen_Incertae_sedis*, and *Natipusillaceae_gen_Incertae_sedis* were closely associated with the J monoculture.

### 3.4. Responses of Key Microbial Taxa to Different Planting Patterns

**Figure 5:**
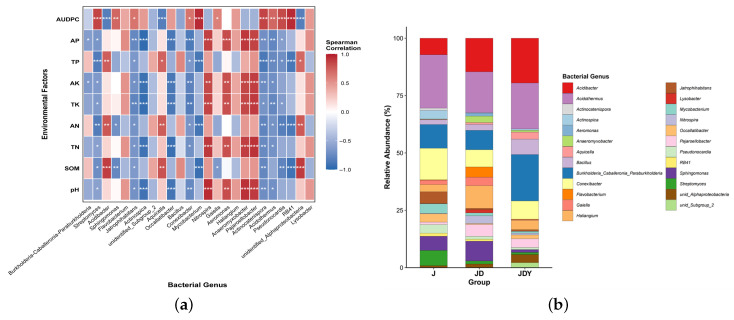
Correlation analysis of key bacterial taxa with environmental factors (including AUDPC) and their relative abundances under different planting patterns. (**a**) Heatmap showing correlations between key bacterial genera and environmental factors. Red and blue boxes indicate positive and negative correlations, respectively. * *p* < 0.05; ** *p* < 0.01; *** *p* < 0.001. (**b**) Stacked bar plot showing the relative abundances of key bacterial taxa under different planting patterns. The height of each bar represents the mean relative abundance of each taxon within the corresponding treatment group.

**Figure 6:**
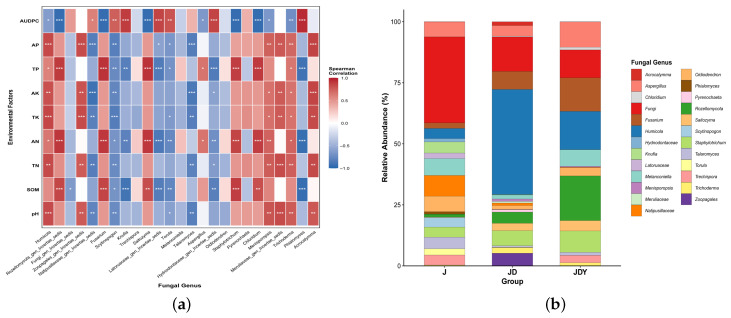
Correlation analysis of key fungal taxa with environmental factors (including AUDPC) and their relative abundances under different planting patterns. (**a**) Heatmap showing correlations between key fungal genera and environmental factors. Red and blue boxes indicate positive and negative correlations, respectively. * *p* < 0.05; ** *p* < 0.01; *** *p* < 0.001. (**b**) Stacked bar plot showing the relative abundances of key fungal taxa under different planting patterns. The height of each bar represents the mean relative abundance of each taxon within the corresponding treatment group.

Correlation heatmaps were used to examine the associations between key microbial taxa and environmental factors, as well as their relative abundances under different planting patterns. SOM and AUDPC, representing soil health and disease severity, respectively, were selected as the primary indicators for correlation analysis (Figure 5 and Figure 6).

Among the key bacterial taxa, SOM showed a highly significant positive correlation with *unidentified_Alphaproteobacteria*, *Aquicella*, and *Acidibacter*, all of which displayed higher relative abundances in the JD and JDY plots, with the highest levels observed in JDY. In contrast, AUDPC was strongly positively correlated with *Streptomyces*, *Mycobacterium*, *Actinocatenispora*, *Sphingomonas*, *RB41*, *Pseudonocardia*, and *Acidothermus*, which were most abundant in the J monoculture. Notably, *Bacillus* and *Burkholderia-Caballeronia-Paraburkholderia* were significantly enriched in JDY, and both taxa were positively correlated with SOM but negatively correlated with AUDPC (Figure 5 and Table S1).

Among the key fungal taxa, SOM showed a highly significant positive correlation with *Chloridium*, *Saitozyma*, *Rozellomycota_gen_Incertae_sedis*, *Fusarium*, and *Staphylotrichum*. These genera displayed higher relative abundances in the JD and JDY plots, with JDY exhibiting the highest levels. AUDPC was strongly positively correlated with *Knufia*, *Scytinopogon*, *Torula*, *Latoruaceae_gen_Incertae_sedis*, *Hydnodontaceae_gen_Incertae_sedis*, and *Phialomyces*, all of which were most abundant in the J monoculture. Notably, *Trichoderma* was significantly enriched in JDY and exhibited a positive correlation with SOM but a highly significant negative correlation with AUDPC (Figure 6 and Table S2).

### 3.5. Functional Prediction of Key Microbial Taxa

**Figure 7:**
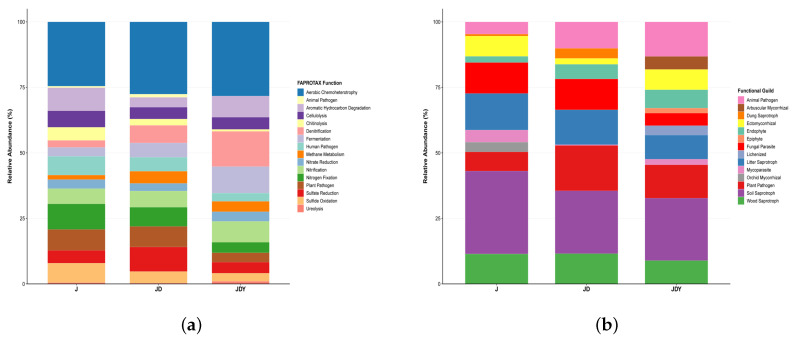
Functional prediction of key soil microbial taxa. (**a**) Stacked bar plot showing the predicted functional potentials of key bacterial taxa based on FAPROTAX. (**b**) Stacked bar plot showing the predicted functional potentials of key fungal taxa based on FUNGuild. The height of each bar represents the relative strength of functional potential.

Functional predictions of the selected key microbial taxa were performed using the FAPROTAX and FUNGuild databases, revealing apparent functional differences among the planting patterns (Figure 7).

A total of 16 functional groups were identified among the key bacterial taxa, with Aerobic Chemoheterotrophy being the dominant category. In the JDY plots, functions related to nitrogen and carbon cycling—such as Denitrification, Fermentation, Nitrification, Methane Metabolism, and Ureolysis—showed high abundance. The JD plots exhibited higher levels of Sulfate Reduction and Methane Metabolism, which are associated with sulfur and carbon cycling. In contrast, the J monoculture primarily exhibited functions associated with environmental modification, such as Chitinolysis and Sulfide Oxidation. Notably, pathogen-associated functions were more abundant in both the J and JD treatments.

Fourteen functional groups were identified among the key fungal taxa, with Saprotrophic guilds (Soil, Wood, and Litter Saprotrophs) being dominant. The JDY plots showed higher abundances of Endophyte, Arbuscular Mycorrhizal, Epiphyte, and Lichenized fungi, the latter three occurring exclusively in JDY, suggesting enhanced soil development and fertility. In the JD plots, Dung Saprotroph, Wood Saprotroph, Fungal Parasite, and Litter Saprotroph were more prominent. The J monoculture exhibited higher abundances of Soil Saprotrophs, Mycoparasites, and Orchid Mycorrhizal fungi.

### 3.6. Co-Occurrence Network Analysis

**Figure 8:**
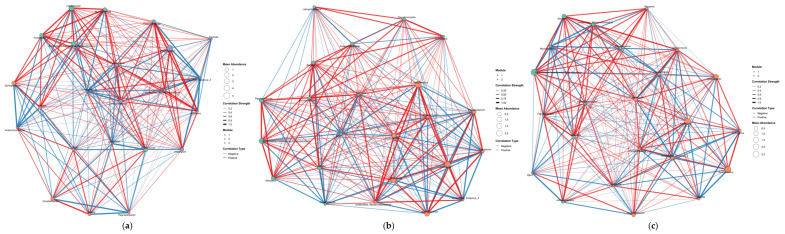
Co-occurrence networks of key bacterial taxa under different planting modes. (**a**) Co-occurrence network of key bacterial taxa in the J planting mode; (**b**) Co-occurrence network of key bacterial taxa in the JD planting mode; (**c**) Co-occurrence network of key bacterial taxa in the JDY planting mode. Red edges indicate positive correlations, while blue edges indicate negative correlations. Thicker edges represent stronger correlations.

**Figure 9:**
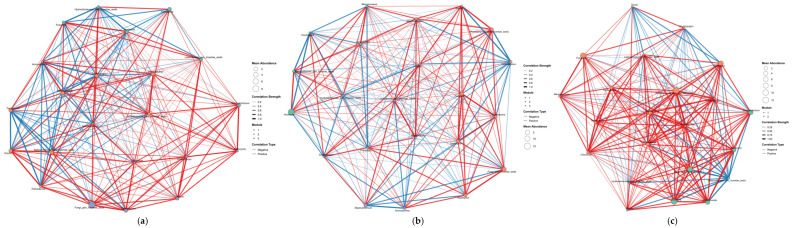
Co-occurrence networks of key fungal taxa under different planting modes. (**a**) Co-occurrence network of key fungal taxa in the J planting mode; (**b**) Co-occurrence network of key fungal taxa in the JD planting mode; (**c**) Co-occurrence network of key fungal taxa in the JDY planting mode. Red edges indicate positive correlations, while blue edges indicate negative correlations. Thicker edges represent stronger correlations.

To elucidate the interaction patterns of microbial communities across different planting modes, co-occurrence networks were constructed from the identified key microbial taxa (Figure 8 and Figure 9).

In the J network, strains such as *Streptomyces*, *Mycobacterium*, *Sphingomonas*, *RB41*, *Pseudonocardia*, and *Acidothermus* are primarily located in the central area of the network diagram; its network betweenness is *Haliangium*; and *Aquicella* and *Acidibacter* are both found in marginal areas. In the JD and JDY networks, as plant species diversity increases, *Aquicella* and *Acidibacter* gradually enter the network’s central region, and the positive correlations between *Burkholderia-Caballeronia-Paraburkholderia* and *Bacillus* gradually strengthen, reaching the highest level in JDY. BCP is strongly positively correlated with *Bacillus*, and core biocontrol bacterial genera are strongly positively correlated with functional bacterial genera, with the interaction relationship showing a clear functional orientation. The Betweenness of the JD and JDY networks is *unidentified_Subgroup_2* and *Lysobacter*, respectively (Figure 8).

The co-occurrence network correlations of key fungal groups and the average abundance of genera showed significant differences. Although the strains that significantly correlated with SOM and AUDPC did not show movement in or out of the network diagram, the number of positive-correlation connections in the JDY network increased significantly. The average abundance of each genus in the JD and JDY networks was significantly higher than that in the J network. The biocontrol fungus *Trichoderma* gradually strengthened its role in all three networks and simultaneously moved toward the network center. The Betweenness in the J network was *Latoruaceae_gen_Incertae_sedis*, while the Betweenness in both the JD and JDY networks was *Scytinopogon* (Figure 9).

## 4. Discussion

The occurrence and severity of plant diseases are not isolated events but are often tightly coupled with specific soil environmental conditions and the associated microbial communities [50]. In this study, we found that compared with the *Dalbergia odorifera* monoculture (J), both the *D. odorifera–Pterocarpus macrocarpus* mixed forest (JD) and the *D. odorifera–P. macrocarpus–Clinacanthus nutans* complex mixed forest (JDY) exhibited significantly reduced anthracnose severity, with the JDY system showing the lowest AUDPC value. Soil physicochemical analyses further revealed that both the JD and JDY planting systems significantly enhanced soil nutrient status. These findings suggest a potential link between soil health and the severity of anthracnose disease. Deng, Yong et al. analyzed microbial enrichment processes in the rhizosphere of plants with different health conditions and demonstrated that soil physicochemical properties are the key drivers determining whether beneficial microorganisms or pathogens are preferentially enriched in the rhizosphere, thereby directly influencing disease development [51]. Zheng, Yanfen et al. further showed that beneficial keystone taxa isolated from disease-suppressive soils serve as crucial biological sources conferring suppressive capacity to the soil [52]. Tao, Chengyuan et al. demonstrated that manipulating soil environmental conditions to enrich indigenous beneficial *Pseudomonas* populations markedly enhances the soil’s ability to suppress soil-borne diseases [53]. To further elucidate the relationships among planting patterns, microbial community assembly, and disease variation, redundancy analysis (RDA) revealed that planting patterns drive microbial community assembly, explaining 79% and 80.3% of the variation in bacterial and fungal community composition, respectively. The primary environmental factors influencing bacterial community composition were SOM, AUDPC, and pH, whereas SOM, TK, AUDPC, and AK were the dominant drivers shaping fungal community structure. The AUDPC vector was oriented opposite to vectors representing SOM and other soil health indicators (angle > 90°), indicating a negative correlation. It also pointed in the opposite direction of the JD and JDY samples, while aligning with the J cluster. This visually demonstrates that poorer soil health is associated with higher disease severity. Mixed-species planting enhances litter diversity and root complementarity, thereby improving soil structure and nutrient availability, promoting the accumulation and redistribution of key elements such as carbon and nitrogen, and creating favorable conditions for microbial community development. These improvements enhance soil fertility and overall soil health, ultimately reducing the incidence of plant diseases [8,54].

When plants are subjected to pathogen stress, their roots may release specific “cry-for-help” signals that actively recruit or enrich beneficial microorganisms with antagonistic, growth-promoting, or colonization-resistance functions, thereby establishing disease-suppressive microbial communities and enhancing systemic plant resistance [55]. Although root exudates were not directly measured in this study, the synchronous changes observed between microbial community structure and disease indicators are consistent with this framework. To identify beneficial microorganisms whose abundance is driven by planting patterns, this study selected the 25 key genera exhibiting the strongest responses to environmental drivers based on RDA. Correlation with environmental factors and differential abundance analyses revealed that the enrichment of these genera differed significantly across planting patterns (Tables S1 and S2). Genera such as *Aquicella*, *Acidibacter*, *Bacillus*, *Burkholderia-Caballeronia-Paraburkholderia*, *Chloridium*, *Saitozyma*, *Staphylotrichum*, and *Trichoderma*, which were positively correlated with SOM and negatively correlated with AUDPC, exhibited significantly higher abundance in the JDY system compared to the J system. In the JD system, genera including *Aeromonas*, *Nitrospira*, *Anaeromyxobacter*, *Zoopagales_gen_Incertae_sedis*, *Meruliaceae_gen_Incertae_sedis*, and *Humicola*, which were positively correlated with soil pH, TK, and AK, were significantly enriched. These beneficial genera may enhance the health of *D. odorifera* through multiple mechanisms. (1) One is directly antagonizing pathogens or inducing resistance, thereby reducing anthracnose incidence. *Trichoderma*, a well-known biocontrol fungus, can directly antagonize a wide range of foliar pathogens, significantly decreasing leaf disease incidence [56]. *Bacillus* has been extensively demonstrated to control forest diseases via the production of antimicrobial compounds, niche competition, and induction of systemic resistance in plants, and it has been widely applied in the biocontrol of tree pathogens [57]. Certain members of *Burkholderia-Caballeronia-Paraburkholderia* can suppress pathogenic fungi through the production of volatile sulfur compounds and other metabolites [58]. Notably, *Burkholderia* strains have been repeatedly isolated from *D. odorifera* root nodules and have been confirmed to form effective symbioses, significantly promoting plant growth and nitrogen accumulation, representing a key symbiotic nitrogen-fixing resource for *Dalbergia odorifera* [59]. Related strains, such as *Paraburkholderia*, can colonize multiple leguminous hosts and possess multifaceted functions, including growth promotion and stress resistance, demonstrating high ecological adaptability and considerable application potential [60]. Nitrogen-fixing bacteria are ubiquitously present and play essential roles in nodule development and function. Through symbiotic interactions with plants, they mediate biological nitrogen fixation from atmospheric N_2_, constituting a key microbial community for nodule formation and functional nitrogen fixation [61]. Extensive studies have demonstrated that nitrogen-fixing bacteria include not only the classical *Rhizobium* but also various non-rhizobial diazotrophs. These bacteria collectively enhance nodule development and nitrogen-fixation efficiency through multiple mechanisms, such as modulating plant hormones and promoting mineral uptake, further highlighting the diversity and ubiquity of nitrogen-fixing bacteria in nodule formation [62,63]. (2) Another mechanism is promoting soil nutrient cycling and environmental improvement, thereby indirectly reducing disease incidence. *Acidibacter* and *Nitrospira* primarily regulate soil pH and nitrogen cycling, optimizing the rhizosphere microenvironment and indirectly enhancing plant health and disease resistance [64]. *Aeromonas* can improve soil structure by secreting extracellular polysaccharides, thereby promoting plant growth, nutrient uptake, and enhancing soil health and crop productivity [65]. *Saitozyma* secretes extracellular enzymes, such as lipases, that participate in the decomposition of lipids and organic matter, promoting soil organic carbon mineralization and cycling [66]. *Anaeromyxobacter*, as a typical metal-reducing bacterium, along with *Aquicella*, plays an important role in iron cycling and the turnover of multiple soil nutrients [67,68]. (3) A further mechanism is promoting plant growth and enhancing stress tolerance. *Humicola* and *Staphylotrichum* can improve plant growth and confer greater resilience to environmental stressors [69,70]. In summary, the microbial communities enriched under the JDY system exhibit a more diverse set of functional pathways, combining direct pathogen antagonism, growth promotion, environmental amelioration, and enhanced plant resistance. In contrast, the communities enriched in the JD system primarily maintain *D. odorifera* health by promoting nutrient cycling and enhancing plant resilience.

Functional prediction further revealed a significant differentiation in the primary ecological functions of microbial communities across planting patterns, particularly among fungi. The JDY system uniquely exhibited three functional types: Arbuscular Mycorrhizal, Epiphytic, and Lichenized. Numerous studies have confirmed that these functions collectively improve soil and environmental conditions through multiple mechanisms, including enhancing plant nutrition and resistance, directly antagonizing pathogens, and producing novel antimicrobial compounds, thereby significantly enhancing plant disease resistance and ecosystem stability [71,72,73]. In bacterial functional predictions, the JDY system showed that the bacterial community was more oriented toward carbon and nitrogen cycling. In contrast, the JD system was more closely associated with sulfur and carbon cycling. Previous studies have demonstrated that diverse mixed-species forests can significantly enhance soil nutrient cycling rates, thereby improving plant resistance to diseases [74,75]. In contrast, the monoculture (J) community primarily maintained basic soil environmental functions yet exhibited higher pathogen-related functional potential, indicating an elevated latent risk of disease. It should be noted that the function predictions in this study, based on taxonomic annotation, have inherent limitations. They primarily rely on mapping known taxa to known functions, whereas microbial functions are often strain-specific and environmentally dependent; even within the same genus, members may include pathogenic, nonpathogenic, and antagonistic strains. To address this limitation, future studies could integrate metagenome-assembled genomes (MAGs) to dissect the composition of functional genes and metabolic pathways within microbial communities. The application of MAGs not only greatly expands genomic resolution for uncultivated microbes but also unveils their metabolic potential and ecological roles in key biogeochemical cycles, such as carbon, nitrogen, and sulfur, providing a solid foundation for understanding microbial contributions to environmental stability and ecosystem services [76]. Additionally, RNA-seq allows real-time capture of functionally active genes and metabolic pathways during disease onset and progression. Advanced approaches such as dual RNA-seq can simultaneously analyze pathogen and host transcriptome dynamics, revealing pathogen gene regulation during infection and molecular interactions with the host, providing a powerful tool to unravel disease mechanisms and identify novel control targets [77]. Therefore, integrating multi-omics approaches that combine MAGs and RNA-seq will facilitate comprehensive elucidation of microbial community functions from the genomic to the transcriptomic levels, encompassing disease progression, nutrient cycling, and other ecological processes.

Co-occurrence network analysis further validated this ecological optimization pathway from a microbial interaction perspective. As the stand structure shifted from J to JDY, the fungal network exhibited a clear transition from an unstable community to an efficient decomposition system and ultimately to a robust defense-oriented assemblage. In the J network, the core hub was the functionally unknown *Latoruaceae_gen_Incertae_sedis*, suggesting that the system’s stability and functional regulatory capacity are difficult to predict. In the JD network, the central taxa shifted toward *Scytinopogon*, a lignin-degrading fungus, suggesting a transition toward more efficient nutrient cycling [78]. In the JDY network, *Scytinopogon* continued to occupy the central position, while functional linkages involving potent biocontrol fungi, such as *Trichoderma*, became increasingly strengthened. A comprehensive review by Sood, Monika et al. demonstrated the diverse biological functions of *Trichoderma* spp., confirming its role as a versatile biocontrol agent [79]. The JDY system formed a mature, stable network that integrates both efficient decomposition capacity and strong pathogen suppression. Changes in the bacterial networks revealed a functional shift in the microbial community from “internal competition” to “cooperative defense”. In the J system, the core hub was the predatory bacterium *Haliangium*. Previous studies have demonstrated that *Haliangium*, as a unique functional taxon with active predatory capabilities, often dominates ecological interactions characterized by predation and competition [80]. Therefore, the J network is inferred to be primarily governed by competitive interactions. In the JD system, the core taxon shifted to the functionally uncharacterized unidentified_Subgroup_2, suggesting that the microbial community may be undergoing structural reorganization. In the JDY stage, the biocontrol bacterium *Lysobacter* became the central hub. It formed positive cooperative associations with *Flavobacterium*, *Gaiella*, and other taxa, thereby establishing a highly effective defense network centered on pathogen suppression. Drenker, Christian et al. demonstrated that *Lysobacter* exhibits broad-spectrum antagonistic activity against diverse plant-pathogenic bacteria, fungi, and oomycetes and provides disease control efficacy comparable to that of conventional chemical pesticides across multiple crop species [81]. Nishioka, Tomoki et al. confirmed, through rhizosphere isolation and inoculation experiments, that *Flavobacterium* can significantly suppress cucumber wilt disease, and microscopic imaging further revealed its ability to inhibit the proliferation of pathogenic fungi in soil [82]. Wei, Chengjian et al. verified, through multi-omics analyses and correlation experiments, that *Gaiella* is closely associated with the synthesis of antimicrobial metabolites and plays an important role in shaping disease-suppressive microbial networks in healthy soils [83]. Moreover, the strong positive association between *Burkholderia–Caballeronia–Paraburkholderia* and *Bacillus* indicates a functionally oriented interaction between core biocontrol taxa and functional microbial groups, collectively contributing to the maintenance of soil health and ecological balance.

Notably, in the JDY system—which exhibited the most substantial disease-suppressive effect—*Fusarium* showed a relatively high abundance. Although *Fusarium* is commonly regarded as a typical soil-borne pathogenic genus, studies have demonstrated that numerous non-pathogenic and even biocontrol-competent strains exist within this genus. These strains can act as antagonistic or symbiotic fungi, contributing to the development of suppressive soils [84]. Therefore, the elevated abundance of *Fusarium* in JDY soils more likely reflects the enrichment of non-pathogenic or functionally specialized members of the genus, rather than an increased risk of disease. Future studies integrating MAGs with RNA-seq-based multi-omics approaches may elucidate the ecological significance underlying the high abundance of *Fusarium*, from genomic potential to functional expression. Moreover, isolation, pathogenicity assays, and functional validation will be essential for dissecting the specific ecological roles of these *Fusarium* members.

## 5. Conclusions

By characterizing the responses of soil microbiomes to anthracnose under different planting patterns of *D. odorifera*, this study demonstrates that planting pattern is a key driver of soil microbial community reassembly and a significant determinant of disease outcomes. Mixed forests (JD), particularly the composite mixed system (JDY), substantially improved soil physicochemical properties and enhanced nutrient cycling processes. These improvements reshaped the soil microbiome’s functional profiles and gave rise to unique beneficial functional groups, such as arbuscular mycorrhizal fungi. At the same time, beneficial genera such as *Burkholderia-Caballeronia-Paraburkholderia*, *Bacillus*, and *Trichoderma* were significantly enriched, and the microbial communities across the three planting modes evolved from competitive to collaborative defense, forming a steady-state network that integrates nutrient decomposition and pathogen inhibition functions. In summary, the composite mixed planting system modulates soil properties and promotes the enrichment of key functional microbial taxa, thereby achieving ecological suppression of anthracnose. In the establishment or restoration of *D. odorifera* plantations, adopting the JDY composite mixed system and targeting beneficial taxa enriched therein—such as *Burkholderia-Caballeronia-Paraburkholderia* and *Trichoderma*—could serve as strategic indicators of soil health and disease-suppression potential. The research provides new ideas and theoretical support for guiding the ecological management of rare tree species, the establishment of artificial forests, and the ecological prevention and control of diseases.

## Figures and Tables

**Table 1:** Basic information of *the D. odorifera* plantation sample plot.

Plot Number	Planting Pattern	Species Composition
J	Monoculture planting	*D. odorifera*
JD	Binary mixed planting	*D. odorifera* + *Pterocarpus macrocarpus*
JDY	Ternary composite mixed planting	*D. odorifera* + *P. macrocarpus* + *Clinacanthus nutans*

J: *D. odorifera* pure forest; JD: *D. odorifera* and *P. macrocarpus* mixed forest; JDY: *D. odorifera* and *P. macrocarpus* mixed forest with interplanted *C. nutans*.

**Table 2:** Physicochemical enzyme activity properties of soil in different planting patterns of *D. odorifera*.

Plot Number	pH	SOM (g/kg)	TN (g/kg)	AN (mg/kg)	TK (g/kg)	AK (mg/kg)	TP (g/kg)	AP (mg/kg)
J	4.30 ± 0.01 c	7.45 ± 0.21 c	0.11 ± 0.00 c	30.25 ± 1.33 c	2.06 ± 0.03 c	37.02 ± 0.90 c	0.27 ± 0.04 c	19.66 ± 1.92 c
JD	5.60 ± 0.11 a	15.16 ± 0.14 b	0.31 ± 0.01 a	37.31 ± 0.77 b	3.16 ± 0.04 a	57.45 ± 0.85 a	0.37 ± 0.04 b	31.54 ± 0.67 a
JDY	5.17 ± 0.03 b	16.77 ± 0.18 a	0.20 ± 0.02 b	44.34 ± 1.09 a	2.22 ± 0.02 b	48.00 ± 0.55 b	0.52 ± 0.02 a	27.51 ± 0.67 b

Data in the table are expressed as mean ± standard deviation (SD). One-way ANOVA was used to analyze the differences between treatments, and the Tukey test was used for post hoc comparison to identify differences between group means. Different letters within each column indicate statistically significant differences (*p* < 0.05); the same letter denotes no significant difference between treatments, while different letters indicate significant differences. A longitudinal analysis was performed on the data. The following abbreviations are used: SOM, soil organic matter; TN, total nitrogen; AN, available nitrogen; TK, total potassium; AK, available potassium; TP, total phosphorus; AP, available phosphorus.

**Table 3:** Sequencing data and community diversity review.

Group Name	Bacterial Taxa			Fungal Taxa		
	J	JD	JDY	J	JD	JDY
Coverage	1.00 ± 0.000 a	1.00 ± 0.000 a	1.00 ± 0.000 a	1.00 ± 0.000 a	1.00 ± 0.000 a	1.00 ± 0.000 a
Chao	192.5 ± 1.000 b	208 ± 12.437 a	164 ± 1.155 c	124 ± 2.160 b	154.25 ± 6.702 a	133.5 ± 5.972 b
Simpson	0.968 ± 0.001 b	0.976 ± 0.002 a	0.961 ± 0.002 c	0.908 ± 0.023 ab	0.863 ± 0.032 b	0.911 ± 0.008 a
Shannon	4.087 ± 0.022 b	4.332 ± 0.034 a	3.932 ± 0.020 c	3.269 ± 0.139 a	2.9985 ± 0.172 ab	2.924 ± 0.100 b

The table displays data as mean ± standard deviation, with a one-way ANOVA used to analyze group differences, followed by Tukey’s test to separate statistically significant means. Different letters within each row indicate statistically significant differences (*p* < 0.05); the same letter denotes no significant difference between treatments, whereas different letters indicate significant differences. Row-wise analysis is performed on the data.

## Data Availability

The original contributions presented in this study are included in the article/Supplementary Material. Further inquiries can be directed to the corresponding author.

## Supplementary Materials

The following supporting information can be downloaded at https://www.mdpi.com/article/10.3390/microorganisms13122876/s1, Figure S1. Principal coordinates analysis (PCoA) based on genus-level compositions of bacterial (a) and fungal (b) communities revealed distinct overall community structures among the different planting systems. Table S1. Analysis of differences in the abundance of key bacteria in soil under different planting modes. Table S2. Analysis of the differences in the abundance of key fungi in soil under different planting methods.
