# Supplementary material for: Responses of Soil Microbial Communities and Anthracnose Dynamics to Different Planting Patterns in Dalbergia odorifera
Source: Microorganisms. 2025 Dec 18;13(12):2876. doi: 10.3390/microorganisms13122876 (PMC12735525; doi:10.3390/microorganisms13122876)
Supplement: Supplementary file 1 [file microorganisms-13-02876-s001.zip › microorganisms-4017568-supplementary.pdf]

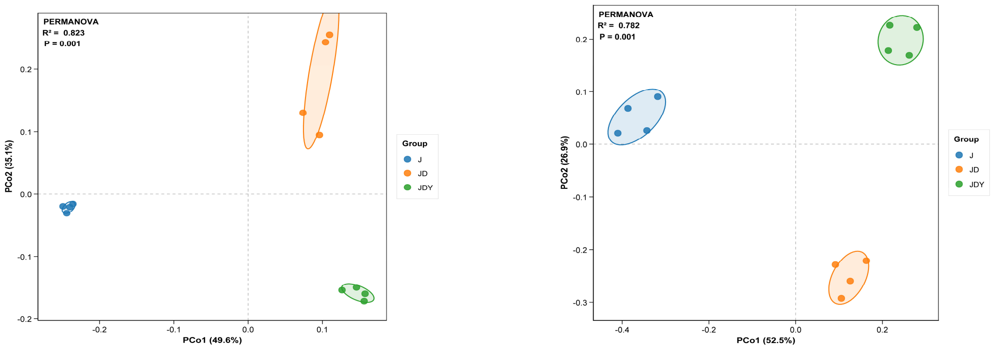

(**a**) (**b**)

Figure S1. Principal coordinates analysis (PCoA) based on genus-level compositions of bacterial (a) and fungal (b) communities revealed distinct overall community structures among the different planting systems. The R^2 and *P*-values shown in the figure represent the statistical test results derived from PERMANOVA.

Table S1. Analysis of differences in the abundance of key bacteria in soil under different planting modes

Genus	J	JD	JDY	Best Group	JD/J	JDY/J	*P*-value
Aeromonas	0.0000±0.0000b	0.1768±0.1271a	0.0000±0.0000b	JD	Inf	1.00×	0.005
Actinospica	0.8384±0.1081a	0.0339±0.0163c	0.0797±0.0117b	J	24.71×↓	10.52×↓	0.007
unidentified_Subgroup_2	0.0239±0.0055b	0.0056±0.0065c	0.2846±0.0235a	JDY	4.24×↓	11.91×↑	0.007
Occallatibacter	0.8146±0.1163a	0.0568±0.0220c	0.2213±0.0142b	J	14.33×↓	3.68×↓	0.007
Mycobacterium	0.9439±0.0953a	0.1794±0.0187b	0.1047±0.0115c	J	5.26×↓	9.02×↓	0.007
Nitrospira	0.0368±0.0047c	0.4681±0.1881a	0.0988±0.0266b	JD	12.73×↑	2.69×↑	0.007
Haliangium	0.7036±0.1152b	1.3722±0.2624a	0.5320±0.0514c	JD	1.95×↑	1.32×↓	0.007
Anaeromyxobacter	0.0264±0.0023c	0.3882±0.0906a	0.1021±0.0205b	JD	14.71×↑	3.87×↑	0.007
Pajaroellobacter	0.2353±0.0370c	0.7188±0.1306a	0.4950±0.0444b	JD	3.06×↑	2.10×↑	0.007
RB41	0.2962±0.0488a	0.1158±0.0417b	0.0012±0.0095c	J	2.56×↓	241.30×↓	0.007
Lysobacter	0.0268±0.0041b	0.1864±0.1791a	0.0003±0.0006c	JD	6.96×↑	88.26×↓	0.007
Actinocatenispora	0.2238±0.0228a	0.0006±0.0012b	0.0000±0.0000c	J	368.32×↓	NA	0.009
Flavobacterium	0.0400±0.0043b	0.6231±0.4436a	0.0244±0.0080c	JD	15.60×↑	1.64×↓	0.01
Burkholderia-Caballeronia-Paraburkholderia	2.3099±0.2350b	1.1720±0.8406c	2.6372±0.3126a	JDY	1.97×↓	1.14×↑	0.015
Streptomyces	1.4912±0.0857a	0.1879±0.0652b	0.1215±0.0515c	J	7.94×↓	12.28×↓	0.015
Bacillus	0.4659±0.0722b	0.3616±0.1006c	0.8737±0.1990a	JDY	1.29×↓	1.88×↑	0.015
Acidibacter	1.6097±0.1196c	2.0305±0.4070b	2.5447±0.0784a	JDY	1.26×↑	1.58×↑	0.018
Aquicella	0.0442±0.0062c	0.1122±0.0599b	0.4030±0.0823a	JDY	2.54×↑	9.12×↑	0.018
Gaiella	0.4222±0.0198b	0.5130±0.1042a	0.0412±0.0064c	JD	1.22×↑	10.25×↓	0.018
Pseudonocardia	0.8417±0.0631a	0.1759±0.0975b	0.1226±0.0090c	J	4.78×↓	6.86×↓	0.018
unidentified_Alphaproteobacteria	0.1674±0.0193c	0.2137±0.0532b	0.4623±0.0190a	JDY	1.28×↑	2.76×↑	0.018
Jatrophihabitans	1.1357±0.0763a	0.0864±0.0141b	0.0926±0.0134b	J	13.14×↓	12.26×↓	0.023
Conexibacter	3.0727±0.2009a	1.0418±0.2386b	1.0378±0.1188b	J	2.95×↓	2.96×↓	0.023

Sphingomonas	1.3779±0.0902a	1.1965±0.4112b	0.1554±0.0364c	J	1.15×↓	8.86×↓	0.025
Acidothermus	5.1982±0.2705a	2.4756±0.6826c	2.6135±0.2116b	J	2.10×↓	1.99×↓	0.025

Values are presented as mean ± standard deviation. Different letters indicate significant differences among groups, whereas identical letters denote no significant differences (Kruskal–Wallis test followed by Dunn's post hoc comparison, $P < 0.05$). The "Best group" refers to the group with the highest mean abundance. Fold change represents the ratio of abundance between groups. ↑ Indicates an increase relative to the J group, whereas ↓ indicates a decrease relative to the J group. "Inf" denotes an infinite fold change (a non-zero value divided by zero), while "NA" indicates not applicable (zero divided by a non-zero value). Values should be compared horizontally across groups.

Table S2. Analysis of the differences in the abundance of key fungi in soil under different planting methods

Genus	J	JD	JDY	Best Group	JD/J	JDY/J	*P*_value
Zoopagales_gen_Incertae_sedis	0.0000±0.0000b	1.8306±1.2612a	0.0000±0.0000b	JD	Inf	1.00×	0.005
Meruliaceae_gen_Incertae_sedis	0.0000±0.0000b	0.2378±0.0562a	0.0000±0.0000b	JD	Inf	1.00×	0.005
Rozellomycota_gen_Incertae_sedis	0.3431±0.1502c	1.6189±0.4770b	12.5114±4.9150a	JDY	4.72×↑	36.47×↑	0.007
Fusarium	0.6410±0.3348c	2.6020±1.3568b	9.4092±2.5356a	JDY	4.06×↑	14.68×↑	0.007
Saitozyma	0.0400±0.0635c	1.0471±0.4419b	2.9031±0.9850a	JDY	26.17×↑	72.55×↑	0.007
Staphylotrichum	1.1533±0.4303c	2.1908±0.4509b	5.9897±1.8572a	JDY	1.90×↑	5.19×↑	0.007
Chloridium	0.0022±0.0029c	0.1071±0.0610b	0.7507±0.3934a	JDY	48.15×↑	337.52×↑	0.007
Phialomyces	0.2787±0.1454a	0.0500±0.0321b	0.0035±0.0046c	J	5.58×↓	78.63×↓	0.007
Pyrenochaeta	0.0063±0.0052b	0.3662±0.3783a	0.0000±0.0000c	JD	57.96×↑	NA	0.009
Knufia	1.2747±0.2273a	0.0523±0.0284b	0.0227±0.0234c	J	24.39×↓	56.14×↓	0.012
Talaromyces	1.3318±0.4788a	0.2325±0.0842c	0.8204±0.4070b	J	5.73×↓	1.62×↓	0.012
Scytinopogon	1.1024±0.6331a	0.0019±0.0039b	0.0028±0.0056b	J	569.27×↓	391.00×↓	0.015
Latoruaceae_gen_Incertae_sedis	0.6325±0.1409a	0.0069±0.0069b	0.0007±0.0014c	J	91.28×↓	879.80×↓	0.015
Melanconiella	1.9304±1.0189b	0.5847±0.2221c	4.6338±3.6520a	JDY	3.30×↓	2.40×↑	0.015
Hydnodontaceae_gen_Incertae_sedis	0.3826±0.0643a	0.0044±0.0053b	0.0006±0.0012c	J	86.38×↓	638.66×↓	0.015
Natipusillaceae_gen_Incertae_sedis	0.3829±0.8291a	0.3489±0.6068a	0.1259±0.0686b	J	1.10×↓	3.04×↓	0.018
Trechispora	1.1555±0.3637b	0.0130±0.0127c	2.0789±1.1859a	JDY	88.52×↓	1.80×↑	0.018
Humicola	1.1664±0.5017c	15.1555±5.6021a	10.7161±4.1026b	JD	12.99×↑	9.19×↑	0.021
Torula	0.7119±0.1788a	0.6226±0.0064b	0.0155±0.0108c	J	1.14×↓	45.83×↓	0.021
Menisporopsis	0.0010±0.0020c	0.3471±0.1215a	0.2770±0.1989b	JD	348.82×↑	278.40×↑	0.022
Aspergillus	1.7046±0.5228b	1.5215±0.5981b	7.1006±1.9048a	JDY	1.12×↓	4.17×↑	0.023
Acrocalymma	0.0335±0.0138b	0.5525±0.7500a	0.0336±0.0061b	JD	16.52×↑	1.00×↑	0.023
Trichoderma	0.0939±0.0277c	0.8026±0.2816b	0.8781±0.4450a	JDY	8.54×↑	9.35×↑	0.025

Oidiodendron	1.7776±0.8959b	0.5340±0.1773c	2.3134±0.8659a	JDY	3.33× ↓	1.30× ↑	0.031
Fungi_gen_Incertae_sedis	9.8530±2.7057a	4.9762±1.6015a	7.8116±8.9705a	J	1.98× ↓	1.26× ↓	0.174

Values are presented as mean ± standard deviation. Different letters indicate significant differences among groups, whereas identical letters denote no significant differences (Kruskal–Wallis test followed by Dunn's post hoc comparison, $P < 0.05$). The "Best group" refers to the group with the highest mean abundance. Fold change represents the ratio of abundance between groups. ↑ Indicates an increase relative to the J group, whereas ↓ indicates a decrease relative to the J group. "Inf" denotes an infinite fold change (a non-zero value divided by zero), while "NA" indicates not applicable (zero divided by a non-zero value). Values should be compared horizontally across groups.
